# Root Canal Operation Controversies

**Published:** 1918-01

**Authors:** Emery Jones

**Affiliations:** New Westminster, B. C.


### ROOT CANAL OPERATION CONTROVERSIES

EMERY JONES, D.D.S., NEW WESTMINSTER, B. C.

With so many essayists and clinicians, it is not surprising to find diversified opinions regarding the theory and technic of the several specialties of dentistry. But to the thousands of dentists who attended the National Dental Association meeting in New York, it seems deplorable that there can not be presented more scientific facts and fewer half-proven conclusions, several of which were so widely different as to be almost in direct opposition.

Dr. Elmer S. Best, of Minneapolis, considers it good practice to use sodium potassium compound or sulphuric acid in gaining access, or enlarging fine canals. He also considers that tricresol-formalin can be used to advantage if sealed in the pulp chamber. In filling roots that have previously been infected, he believes it wise to fill beyond the apex, and where possible to encapsulate the end of the root.

In opposition to this theory, Dr. Fred Gethro, of Chicago, emphasized the advisability of not filling any roots beyond the apex. He does not advise the use of acids in enlarging the canals, because of the danger of injury to the apical tissues, and believes the canals can be satisfactorily enlarged with files and barbed broaches.

Dr. Meyer L. Rhein is an earnest advocate of forcing the root filling beyond the foramen providing the apex is denuded of peridental membrane. If the cementum is left exposed to the action of the osteoclasts, absorption may follow. The streptococcus veridans, so commonly found in the granuloma, though incapable of producing pus, produces toxins even more virulent. These organisms may be destroyed by ionization, but others will replace them if there is left a suitable media for their development. Therefore encapsulate the denuded apex with chloropercha and thus make it armor proof.

Dr. Weston A. Price, of Cleveland, states that few of us have ever sterlized a tooth in the mouth. The medicaments commonly used do not sterilize the dentine, much less the cementum. Nine-tenths of the electric current used in ionization is expended on healthy tissue and only one-tenth on the pathological. Yet the majority of essayists speak with great confidence of their favorite methods of "sterilizing" the tooth. He reminds us that a condensing osteoitis may be just as much a source of infection as a rarefying osteoitis. Let us not place too much dependence on our radiographs until we are competent to interpret them. Remember that a positive picture means something if properly interpreted, but a negative showing does not necessarily indicate healthy apical tissue.

Dr. Carl J. Grove, of St. Paul, Minn., has made exhaustive experiments with Tricresol and Formalin in root canals and has proven to a vast number of dentists that such drugs should never be used in the treatment of putrescent pulps, in spite of the fact that Dr. Buckley's friends have so earnestly advocated its use. Dr. Grove also shows by numerous observations that more rarified apical areas follow the overfilling of canals than are observed in the underfilled.

Ionization is also a bone of contention. Dr. Fette, of Cincinnati, and Dr. George C. Sharpe, of Pasadena, Cal., believe it should have a very prominent place in carrying medicaments into the apical tissues and destroying the organisms found there. But the consensus of opinion on Ionization seems to have been well expressed by Dr. Elmer Best, when he said: "Though I am using it every day, I am not prepared, yet, to express an opinion of the results."

I might mention many illustrations of contradictory teaching in other branches of dentistry, but one other will be sufficient. For several years many of us have been earnest students of the Prosthetic teaching of Professor Gysi and Dr. Geo. Wood Clapp, and now when we have carefully mastered that technic, Dr. Dayton Dunbar Campbell, of Kansas City, endeavors to prove that "measurements of the mandible, tracings of the Condyles, the construction of hypothetical triangles, and the use of the face bow are all nonessential in the construction of dentures possessing the highest degree of efficiency." He was ably supported by Dr. Rupert E. Hall, of Chicago, and Dr. Martin Dewey, of Chicago. They claim that the tactile sense of tooth cusps controls or at least guides the movements of the mandible.

Now what shall we conclude from these diversified opinions? Most general practitioners have not the opportunity to make exhaustive experiments in every branch of dentistry. We look to the professors in our colleges, the scientists in the laboratories of our Research Institutes to draw conclusions that will permit us to gather right conceptions. It is mighty important for the preservation of human life that these differences of opinion should cease. We must give much of our clinical findings, but these Laboratories of Research must digest our findings that we may be able to properly assimilate the essentials.

But, whether or not these controversies cease, we must not forget the necessity of individual effort to master the Pathology of Dental Tissues and to perfect our technique; if necessary to do so, each must specialize in his own favored branch. But, on the other hand, we must not specialize until we become too narrow and thus lead to more unsettled controversy. If the mind is too long focused on one objective we lose our proper perspective.

Dr. E. C. Rosenow, Rochester, Minn., made this statement: "I believe that teeth can be devitalized, the roots filled and no infection follow, *but* this operation must not be performed in the careless, wholesale manner of the past. Determine not to devitalize teeth without the deepest consideration, and if you do so, then treat them with the utmost care."

Dr. Thomas B. Hartzell impressed upon us that bacteriologically clean mouths would save thousands of lives each year. The streptococcus viridans is usually found in granuloma of the teeth; the same streptococcus is found in masses in heart lesions; over one hundred and five thousand people died last year in the United States of heart disease, while only ninety-eight thousand died of tuberculosis. Ninety per cent. of these heart lesions are caused by streptococcus viridans. Therefore Dr. Hartzell concludes that if fifteen minutes a day were used from childhood to old age in proper dental attention, then those hundred thousand human beings would live.

Surely, then, we should have no controversies over the essentials, and let this watchword continue—Prophylaxis! Prophylaxis! Prophylaxis! Prophylaxis.—*Oral Health.*

